# Towards next-generation DNA encryption via an expanded genetic system

**DOI:** 10.1093/nsr/nwae469

**Published:** 2024-12-23

**Authors:** Xiaoluo Huang, Zhaohua Hou, Wei Qiang, Honglei Wang, Xiangxiang Wang, Xiaoxu Chen, Xin Hu, Junbiao Dai, Lingjun Li, Guanghou Zhao

**Affiliations:** Shenzhen Key Laboratory of Synthetic Genomics, Guangdong Provincial Key Laboratory of Synthetic Genomics, Shenzhen Institute of Synthetic Biology, Shenzhen Institutes of Advanced Technology, Chinese Academy of Sciences, Shenzhen 518055, China; School of Ecology and Environment, Northwestern Polytechnical University, Xi'an 710129, China; Shenzhen Key Laboratory of Synthetic Genomics, Guangdong Provincial Key Laboratory of Synthetic Genomics, Shenzhen Institute of Synthetic Biology, Shenzhen Institutes of Advanced Technology, Chinese Academy of Sciences, Shenzhen 518055, China; Henan Key Laboratory of Organic Functional Molecule and Drug Innovation, Collaborative Innovation Center of Henan Province for Green Manufacturing of Fine Chemicals, School of Chemistry and Chemical Engineering, Key Laboratory of Green Chemical Media and Reactions, Ministry of Education, Henan Normal University, Xinxiang 453007, China; State Key Laboratory of Antiviral Drug and Pingyuan Lab, Henan Normal University, Xinxiang 453007, China; School of Ecology and Environment, Northwestern Polytechnical University, Xi'an 710129, China; School of Ecology and Environment, Northwestern Polytechnical University, Xi'an 710129, China; School of Ecology and Environment, Northwestern Polytechnical University, Xi'an 710129, China; Shenzhen Key Laboratory of Synthetic Genomics, Guangdong Provincial Key Laboratory of Synthetic Genomics, Shenzhen Institute of Synthetic Biology, Shenzhen Institutes of Advanced Technology, Chinese Academy of Sciences, Shenzhen 518055, China; Shenzhen Branch, Guangdong Laboratory of Lingnan Modern Agriculture, Genome Analysis Laboratory of the Ministry of Agriculture and Rural Affairs, Agricultural Genomics Institute at Shenzhen, Chinese Academy of Agricultural Sciences, Shenzhen 518000, China; Henan Key Laboratory of Organic Functional Molecule and Drug Innovation, Collaborative Innovation Center of Henan Province for Green Manufacturing of Fine Chemicals, School of Chemistry and Chemical Engineering, Key Laboratory of Green Chemical Media and Reactions, Ministry of Education, Henan Normal University, Xinxiang 453007, China; State Key Laboratory of Antiviral Drug and Pingyuan Lab, Henan Normal University, Xinxiang 453007, China; School of Ecology and Environment, Northwestern Polytechnical University, Xi'an 710129, China

**Keywords:** multilevel DNA encryption, unnatural base pair, Codec algorithm, DNA data storage

## Abstract

Information encryption based on DNA data archiving, referred to as DNA encryption, has been advocated for decades and has become highly appealing owing to its remarkable advantages, e.g. high storage capacity, complexity and programmability. Early DNA encryption schemes primarily leveraged the natural four-letter genetic alphabet for data storage, with message-storing DNA sequences easily decrypted by routine DNA sequencing, which is consequently vulnerable to attack and faces severe security challenges. Here, an unnatural base pair (UBP), dNaM-dTPT3, was introduced into the message and/or index DNA sequences, which can be stored either *in vitro* or *in vivo*; this approach achieved the bioorthogonal encryption of ‘secret’ messages, where message DNAs could be selectively, faithfully and readily retrieved or read exclusively in the presence of unnatural bases. Furthermore, a separative computational algorithm, named IM-Codec, was developed to encrypt the data into a ‘key sequence’ and an ‘information sequence’ through UBP insertion. Finally, a UBP-based multilevel DNA encryption approach was developed and validated for data encryption and decryption. The employment of the UBP expanded genetic system for data encryption should provide valuable solutions for archiving highly confidential data and thus usher in a new era of DNA encryption.

## INTRODUCTION

With the rapid development of information technology (IT) and its applications in almost all aspects of our lives, human society is facing severe challenges in exploiting novel solutions for simultaneously improving information storage density and security [1–4]. Among the various promising storage formats, ranging from storage in quantum bits to the metabolome, DNA has emerged as an unparalleled material due to its ultrahigh storage density, portability, biological compatibility, low energy consumption and longevity [5–8]. Since late in the last century, Joe Davis *et al.* and Clelland *et al.* independently demonstrated a landmark scheme for storing and concealing DNA-encoded messages, opening a new era of DNA-based information storage and encryption [6,9]. In the past decade, DNA data storage has made rapid progress in terms of foundational technologies, including advanced methods for efficiently encoding digital information into nucleotide sequences and synthesizing it (writing), the use of various media to organize it for long-term preservation (storage), innovative solutions for selectively retrieving it (random access), and novel approaches for faithfully reading and converting it back to digital data (decoding) [10–18]. Thus far, DNA data storage has been demonstrated to have great potential, and its practical applications are becoming increasingly apparent [5–8,12,19–21]. In parallel, to enhance the security of DNA information, various advanced encryption strategies were developed by complicating the classic DNA encryption approach through incorporating more complex and misleading fake DNAs and concealing or randomizing DNA synthesis design, or exploiting novel DNA structures to store and secure the information [4,22,23]. Nevertheless, these DNA-based strategies are generally restricted to exploiting the natural two-base-pair genetic alphabet (A–T and G–C) to implement general storage applications, whose information could potentially be read or decrypted by the routine sequencing method and that consequently suffers from severe security problems [5–7,22,24,25]. To circumvent the direct sequencing threat, some elegant designs have been previously proposed by exploiting specific DNA interactions or reactions to encrypt the information, but issues such as a requirement for well-controlled interaction or reaction conditions, as well as ensuring data integrity, continue to pose challenges [26,27]. For instance, the bisulfite-mediated C-to-U conversion has been employed to transform bits encoded by C nucleobases and thus allow for DNA encryption [27]. However, such conversion requires harsh conditions (acidic and high temperature), which would lead to DNA degradation and loss of DNA-encoded information [28,29]. In light of these challenges, there is a critical need for the development of a method that not only achieves a high standard of encryption, but which also ensures the integrity and reliable retrieval of the DNA-encoded data.

Unnatural base pairs (UBPs), formed between two synthetic nucleotides, function alongside their natural two-base-pair counterparts to expand the genetic alphabet, allowing the fundamental innovation of canonical DNA molecules with man-made building blocks [30,31]. Over the past two decades, a family of UBPs has been developed, with dNaM-d5SICS (or the advanced version dNaM-dTPT3) being a particularly attractive pair that is well-suited for use with natural base pairs to encode, propagate and retrieve expanded genetic information both *in vitro* and *in vivo* [31–35]. Notably, reading the DNA containing the NaM-TPT3 UBP with conventional sequencing technologies is challenging because the Sanger sequencing signal terminates after the dNaM-dTPT3 site [35,36]. Recently, an emerging approach of translating dNaM-dTPT3 into natural pairs of either G-C or T-A via polymerase chain reaction (PCR) assays depending on the presence or absence of the bridge base isoTAT was established, making it feasible to read NaM-TPT3 pairs simply by Sanger sequencing [35]. Although developments in these technologies to allow UBPs to expand the genetic alphabet provide unprecedented opportunities and convenience in revolutionizing DNA-based applications, the potential of UBPs in the field of information storage remains to be explored.

Multilevel encryption, achieved by integrating multiple encryption schemes, is currently a key technology for silicon-based data storage that enables highly secure data manipulation. However, no comprehensive multilevel encryption strategy for DNA-based data storage has been developed [22,24,25]. In this study, we repurposed UBP technology to implement a multilevel encryption scheme to improve the security of DNA-stored data. First, we improved the utility of DNA cryptography to better secure DNA-encoded information by adding UBPs to a meaningful DNA strand, which can be either sequenced or not by adding or not adding isoTAT, the bridge base that allows NaM-TPT3 to be smoothly sequenced by Sanger-sequencing after base conversion. In addition, UBP technology was used to achieve orthogonality between message DNA containing UBPs and natural junk DNA; this approach promises to improve upon the classical DNA steganography method, which generally relies on a pair of PCR primers (indexes) to extract a ‘secret message’ from a large amount of junk DNA, but which suffers from inherent limitations such as cross-interaction between primers and similar off-target sequences and limited variability of primer sequences [9,22,37,38]. Finally, we adopted a separative computational scheme to further improve information security by developing a novel codec algorithm, IM-Codec, which assigns DNA nucleotides into a set of ‘information nucleotides’ and another set of ‘key nucleotides’, similar to the traditional ‘tiger-shaped tally’ used for ancient troop movements. Overall, a UBP-enabled multilevel DNA encryption scheme that is well-suited to work with the variety of tools developed for contemporary natural DNA-based storage systems is presented, which could serve as a versatile system to better secure DNA information.

## RESULTS

### DNA cryptography by the UBP

**Figure 1.**
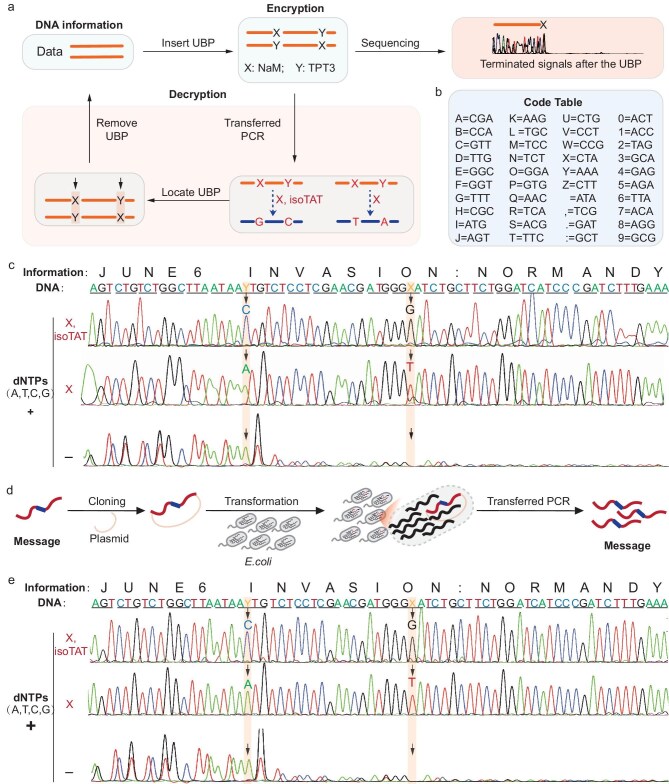
DNA cryptography by the UBP. (a) Illustration of encryption and decryption of information DNA by the UBP. To encrypt the DNA-encoded information, the UBP was inserted into the message DNA via synthesis or PCR using primers containing the UBP, which would lead to the sequencing corruption after the UBP insertion site; to further decrypt the information, the UBP dNaM-dTPT3 (or X-Y) could be converted into either G-C or T-A via transformed PCR by the addition or not of isoTAT in the PCR reactions to enable the UBP to be sequenced, located and finally removed to obtain the final DNA sequences, which could be further decoded according to the code table. (b) Code table used to encode a message in DNA. (c) Sanger sequencing of the PCR products with the addition or not of unnatural bases in the PCR reactions. (d) Illustration of UBP-enabled encryption and decryption of information DNA stored in vivo. The message DNA inserted with UBPs was cloned into an *E. coli* plasmid and transformed into *E. coli* for storage. Further, the information DNA could be PCR amplified from the *E. coli* strains and decrypted as shown in (a). (e) Sanger sequencing of the products obtained by PCR amplification of DNAs stored *in vivo* with the addition or not of unnatural bases in the PCR reactions.

In classical DNA cryptography, DNA sequences containing secret messages are constituted by the natural two-base-pair genetic alphabet (T-A and G–C) and can be easily read or decrypted by the prevalent Sanger-sequencing method. By contrast, message DNAs containing UBPs would generate terminated or corrupted signals by Sanger-sequencing (Fig. 1a) or other advanced technologies including second-generation and third-generation sequencing (Fig. S1), providing an additional safeguard for secret messages. Furthermore, the unnatural base pair dNaM-dTPT3 (or X-Y) could be readily converted into either G-C or T-A via transformed PCR based on the addition or absence of isoTAT in the PCRs; thereby, DNA sequences of both types of PCR products could be acquired by sequencing and further comparatively analyzed to locate and remove the UBPs, making it feasible to decipher the DNA information (Fig. 1a).

To test this UBP-enabled DNA cryptography strategy, two UBPs were added to a prototypical ‘secret message’ DNA strand, which encodes the D-Day message ‘JUNE6 INVASION: NORMANDY’ with a simple substitution cipher to convert the characters into DNA triplets (Fig. 1b) [9]. As expected, a corrupted sequence was obtained by sequencing this message DNA, demonstrating the effectiveness of UBPs in securing DNA-encoded information (Fig. 1c). To decode this message DNA, the UBPs (X-Y) in it were initially converted into either G-C or T-A via PCR assays simply by adding X and isoTAT or only X into the PCRs [35]. Then, robust signals were achieved by sequencing the resultant PCR products to retrieve the information without detectable errors (Fig. 1c). Similar results were obtained for encrypting and decrypting another piece of DNA (Fig. S2), demonstrating the robustness of this UBP-enabled system for faithfully encrypting and decrypting the information stored in DNA with the expanded genetic alphabet.

In addition to the *in vitro* DNA storage system, as demonstrated in the above experiments, an *in vivo* DNA storage system utilizing living organisms to store and propagate DNA information represents a promising alternative [5–7]. To test the ability of the UBP-enabled system to secure DNA information stored *in vivo*, the above D-Day message DNA inserted with UBPs was cloned in an *Escherichia coli* plasmid and transformed into *E. coli* for storage (Fig. 1d, Note S5). Furthermore, the D-Day message DNA was PCR amplified from the resultant *E. coli* strains, and the PCR products were analyzed via Sanger sequencing. As shown in Fig. 1e, robust signals were acquired by sequencing the PCR products after base conversion, whereas corrupted signals were obtained by sequencing the PCR products without base conversion, highlighting the versatility of this UBP-enabled system for securing information in both *in vivo* and *in vitro* DNA storage systems.

### DNA steganography with UBPs

**Figure 2.**
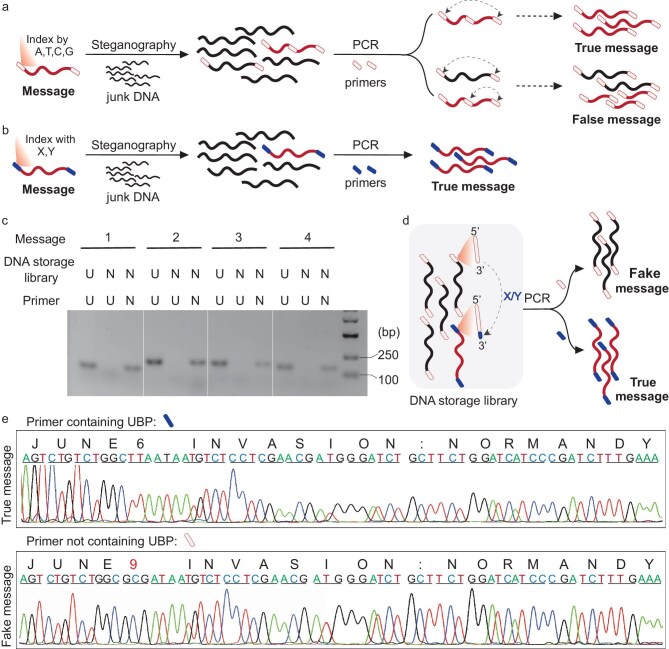
DNA steganography by the UBP. (a) Illustration of classical DNA steganography and its drawbacks due to cross-interactions between index and similar off-target sequences. (b) Illustration of DNA steganography by equipping the primer (or index) sequences with the UBP to circumvent the cross-interaction between index and similar off-target sequences. (c) Gel analysis of PCR products of message DNA libraries. Four message DNAs were flanked by primer sequences comprising either only natural base pairs (Primer N) or both natural and unnatural base pairs with the UBP residing in the 3'terminus (Primer U) (Table S1), and the resultant DNAs were pooled together respectively with four message DNAs containing solely the natural base pairs to construct DNA-storage library ‘N’ and the ones containing UBPs to generate DNA-storage library ‘U’. PCR experiments were conducted with templates and primers as indicated. (d) Illustration of PCR extraction of true or fake message DNA determined by UBP insertion or not in the primers. (e) Sanger sequencing of the PCR-amplified DNAs with two highly similar indexes with the true index harboring only one UBP insertion (upper panel) compared with the fake index (lower panel).

In addition to DNA cryptography, DNA steganography is a mainstream approach for encrypting DNA-stored information; this method classically hides message DNA within a large amount of junk DNA and relies highly on specific PCR primers (indexes) to extract the message DNA from a mysterious DNA storage library [9,24]. As reported in previous studies, the intractable cross-interactions between index and similar off-target sequences can give rise to false messages (Fig. 2a) and accordingly pose great challenges for implementing such DNA steganography [9,22,37,38]. To solve this problem, we designed an orthogonal indexing system by introducing UBPs into the 3′ terminus of the primer sequences to avoid cross-reactions between the index and junk DNAs (Fig. 2b). To test this proposal, we archived four messages into four separate DNAs and flanked them with specific primer sequences comprising either only natural base pairs or both natural and unnatural base pairs, with the UBPs residing in the 3′ terminus (Table S1). Then, these message DNAs containing only natural or both natural and unnatural base pairs were pooled together respectively to generate the DNA-storage libraries ‘N’ (natural) or ‘U’ (unnatural and natural). As shown in Fig. 2c, the four independent pairs of primers with 3′ termini inserted by the UBPs (‘Primer U1-4’) can only index message DNA from the DNA-storage library ‘U’ but not from ‘N’, demonstrating the success of introducing the UBPs to establish an orthogonal indexing system. To further demonstrate the application of this indexing system in DNA steganography, we prepared a DNA storage library containing both true message and fake message DNAs, which could be extracted by two highly similar indexes, where the true index had only one UBP insertion compared with the fake index (Fig. 2d). As shown in Fig. 2e, the true message DNA could be specifically extracted from the storage library using the true index equipped with the UBPs, whereas the fake index without UBP insertion generated the wrong message, revealing the effectiveness of the UBP-enabled orthogonal indexing system in circumventing the cross-interaction between index and junk DNAs in DNA steganography.

### DNA encryption by a separative computational scheme

**Figure 3.**
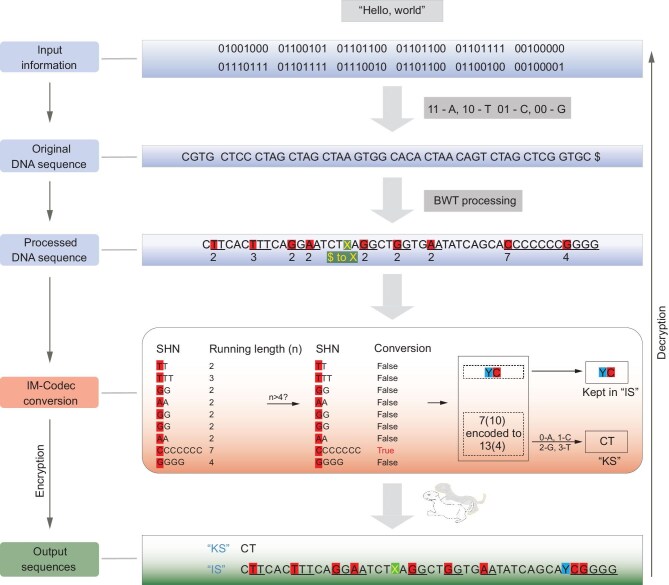
The IM-Codec algorithm pipeline. A predetermined mapping rule is used to first transform the original data into DNA sequence. The encoded DNA sequence ends with an identifying symbol. The encoded DNA sequence is then structured via a BWT processing step into a new sequence with numerous homopolymer runs, with the identifying symbol being changed to the UBP-nucleotide ‘X’. The processed DNA sequences are then encrypted into ‘KS’ and ‘IS’ sequences via IM-codec conversion, which encrypts the single nucleotide repeats. The decryption process is the reverse process of the encryption. ‘7(10) encoded to 13 (4)’ represents the encoding of the decimal ‘7’ into the quaternary ‘13’.

To increase the performance of UBP-based encryption, we developed the IM-Codec algorithm (Fig. 3), which encrypts data into a ‘key sequence’ (KS) and an ‘information sequence’ (IS). Essentially, this approach shortens DNA sequence repetitions and uses ‘marker nucleotides’, such as UBPs, to record the positions of DNA sequence repeats for further decoding. This process begins by converting the binary bits of computer files into A/T/C/G sequences using the defined mapping rules. The sequence is then processed using a Burrows–Wheeler Transform (BWT) sequence transformation to construct longer single-base-repeat sequences and aggregate as many identical nucleotides in the sequence as possible, which generates a new sequence (Fig. S3, Note S1). The BWT is a computational algorithm that reorders a DNA sequence to facilitate the clustering of similar characters, widely employed in next-generation sequencing analysis tools [39–42]. The BWT operation begins by appending a unique end-of-file character ‘$’. This expanded sequence undergoes cyclic right rotations, in which the initial character of the string is shifted to the end, iterating over the entire string until the original sequence is completely rebuilt. These rotations are arranged in lexicographical order to form a matrix, with each row representing a distinct string rotation and the columns filled out in sorted order with the characters from these rotations. The final column of this matrix is retrieved, and identical characters are grouped together owing to rotation sorting, increasing the frequency of repeated characters next to each other. Notably, the BWT is an invertible operation, which ensures that the original string can be reliably reconstructed from the converted version without reducing the data's size, making it be more accessible to run-length control. After BWT conversion, in the new DNA sequence, the ‘X’ base is used to signify the end position of the original DNA sequence. For standard algorithm pipeline, all single nucleotide homopolymers (SNHs) in the sequence with more than four repeats are searched, and the running length is recorded. The relative position of the SNH in the new DNA sequence is preserved during the SNH conversion procedure. The SNH repeat base is used to replace itself, and a marker nucleotide of ‘Y’ bases is inserted before the repeat base to indicate the position of the SNH. The completed sequence is saved as an IS. Moreover, the running duration of each SNH is kept in a separate file for generating the KS. Next, the running lengths are transformed into a series of quaternary numbers, all of which have the same number of digits (the maximum number of digits is the standard) (Note S2). For a quaternary number that is less than the maximum number of digits, zero padding is applied to ensure that every encoded running length comprises an equal base number. The relevant quaternary numbers in the new DNA sequence are then arranged according to the order of the running length-associated SNHs, and KSs are generated using the [0-A, 1-C, 2-G, 3-T] mapping rules. It should be noted that the number of ‘Y’ bases generated by this algorithm can be modified, and the number of ‘Y’ bases in the IS can also be reduced based on actual needs, with the location information of ‘Y’ bases recorded in the KS, to meet different encryption goals. The IM-Codec algorithm is also capable of reconstructing and encrypting any tandem repeat sequence, not only single-base repeats (Fig. S4), improving the versatility of the data encryption. Only if both the KS and IS are obtained can the data stored in the DNA sequence be decrypted, which strongly protects the data from theft. Compared with traditional computational encryption scheme, such as AES, DES, MD5, SHA-1, SHA-384 and SHA-512 [43–49], IM-Codec requires more brute-force efforts to decipher the encrypted message, while the keys have the same level of information content (Note S3). In addition, while encrypting data, this algorithm also encodes the data with a high coding density, allowing the system to store data in the smallest possible space, further boosting data privacy. The algorithm can encode distinct data with the theoretical information density of more than 2 bits/nt by inserting one UBP as the marker nucleotide, and reached the highest theoretical information density of 9.16 bits/nt in tests on video material (Table S2). Notably, the current algorithm converted tandem repeat units with single nucleotide motifs and iterated over four times; however, the lengths of the KS and IS can be adjusted if diverse tandem repeat units were converted. An increase in the repetition count setting of tandem repeat units reduces the original DNA sequence that requires transformation, resulting in a shorter KS and a longer IS. By contrast, converting more types of repeat units, with different numbers of nucleotide composition, may increase the length of the KS while decreasing the length of the IS. Furthermore, by adjusting the total converted tandem repeats and, correspondingly, the numbers of kept unnatural bases, the lengths of the IS and the KS can be adjusted. This will enable diverse data encryption with different storage density (Table S4). With the aid of UBPs, the IM-Codec algorithm can encrypt data with great density and privacy, which is useful for many applications.

### Multilevel DNA encryption enabled by combinatorial approaches

**Figure 4:**
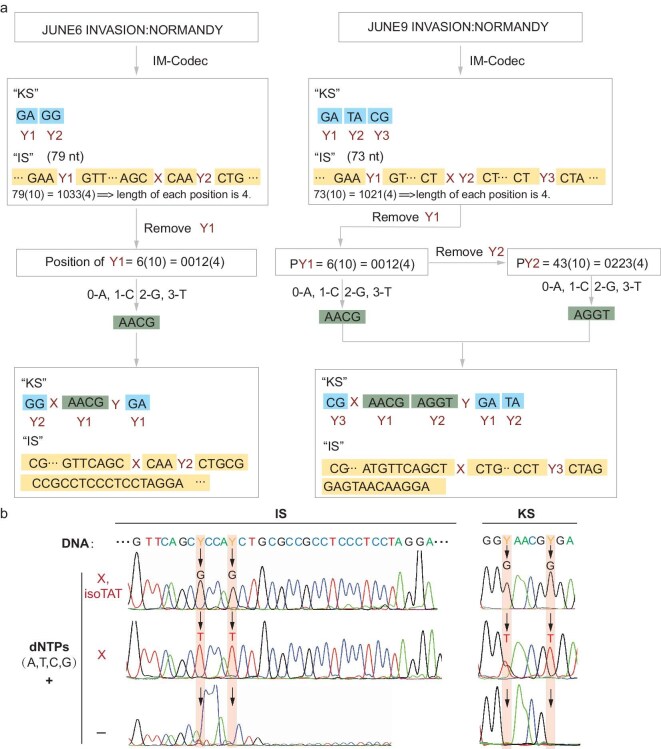
Multilevel DNA encryption enabled by combinatorial approaches. (a) Encoding the D-Day message by IM-codec algorithm. The IM-Codec algorithm firstly translates the true and misleading D-Day message into ‘IS’ and ‘KS’ sequences. We modified the IM-Codec algorithm to insert the UBP bases into both ‘KS’ and ‘IS’ sequences by managing the quantity of ‘Y’ bases in ‘IS’ sequences and altering the structure of ‘KS’ sequences. The ‘Y’ bases that were removed from the original ‘KS’ sequences were then encoded into new ‘KS’ sequences using an adjusted IM-Codec algorithm. The ‘X’ was synthesized as ‘Y’ base and its position information was recorded on the computer disk. (b) IS and KS sequencing peak diagram of information ‘JUNE6 INVASION: NORMANDY’ (see Fig. S5 for the entire sequencing diagram). (10) represents the decimal digital value, while (4) represents the quaternary digital value.

To construct a multilevel DNA encryption scheme for silicon-based data storage, we combined the three approaches above. First, the IM-Codec algorithm is used to encrypt two messages, the D-Day message and a misleading message, into IS and KS sequences (Fig. 4a). Instead of simply inserting UBPs into the IS sequence, we also inserted them into the KS sequence for combined encryption. Therefore, we adjusted the IM-Codec algorithm to remove several Y bases from the originally encoded sequence and put them into the KS sequence through two rounds of conversion. As shown in Fig. 4a, we kept only the second Y base in the encoded IS of the true D-Day message and the third Y base in the fake D-Day message, with other Y bases removed and stored in the KS. To allow additional Y bases that were removed from the original IS sequences, the structure of the originally encoded KS was changed, and its sequence was divided into three sections. The first and third sections stored the running length information for the retained Y base and removed Y base, which were the sequences recorded in the original KS. The second section stored the position information for the removed Y base. Briefly, the position information of the removed Y base was converted into a quaternary number, which was further encoded into the DNA sequence by the mapping rules of [0-A, 1-C, 2-G, 3-T]. The three sections were divided by the insertion of additional ‘Y’ bases. To achieve feasible storage for improving the privacy of information storage, we recorded the position of the ‘X’ base on the computer and synthesized it as ‘Y’ base. By these steps, we obtained the new IS and KS sequences for further chemical synthesis and an ‘X’ base position information recording on the computer disk. Furthermore, these new IS and KS DNA sequences were flanked by index DNA sequences, with the indexes for the true D-Day message having a UBP at the 3′ end and the fake indexes comprising solely the natural genetic alphabet. Then, we synthesized these DNAs and combined them to construct a DNA storage library containing both true and fake messages. As shown in Fig. 4b and Fig. S5a, robust sequencing signals representing the true KS and IS sequences could only be extracted from the storage library after the decryption manipulation of PCR selection using the UBP-equipped indexes and base conversion by the transformed PCR, while the fake KS and IS sequences were acquired by using indexes without UBP insertion (Fig. S5b). The stored messages were further successfully decrypted by the combination of the true KS and IS sequences and recorded ‘X’ base information, by IM-Codec algorithm. Together, our study demonstrates a proof-of-concept multilevel encryption scheme for DNA-stored information.

## DISCUSSION

**Figure 5.**
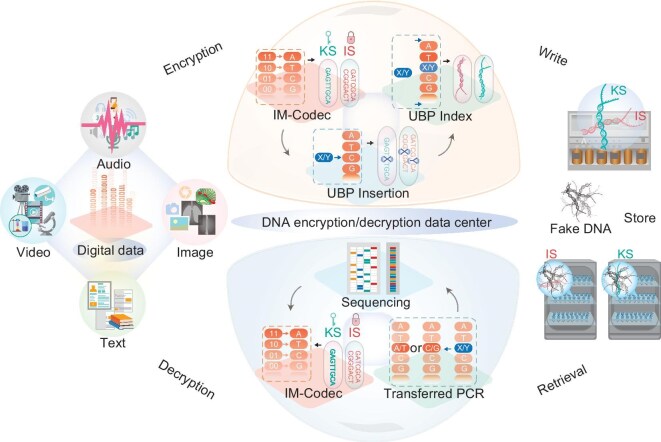
Illustration of the UBP-based multilevel encryption system. A variety of private data, including texts, audio, images and video, were initially encoded into binary sequences, which were then converted into ‘KS’ and ‘IS’ DNA sequences by the IM-Codec algorithm, with UBPs inserted into the sequences and UBP-indexes added to both ends. Chemical DNA synthesis was performed to synthesize these sequences, which could be subsequently hidden within a large amount of fake DNA and kept in separate facilities. UBP-specialized DNA sequencing (featured by using transformed PCR) was used to decrypt the stored DNA, which were subsequently converted to A/T/C/G sequences and decoded back to the binary sequences to acquire the original data.

DNA has been widely studied as a representative next-generation data storage medium that offers ultrahigh storage density, replicability and durability [5–8]. However, in existing DNA storage systems, information security, an extremely crucial issue in modern society, represents a pivotal challenge to be addressed for practical DNA data storage [1,4,22,24,25]. In this study, a multilevel encryption system (Fig. 5) specialized for DNA storage was developed. A customized IM-Codec algorithm was developed to achieve high-density data encryption in which messages are computationally translated into a KS and an IS with UBPs to mark the positions of repeated DNA sequences for further decoding. Furthermore, UBPs were written into message and/or index DNA sequences to implement a bioorthogonal information storage method whereby message DNAs can be selectively, faithfully and readily retrieved or read from highly similar junk DNAs only in the presence of unnatural bases (dNaM, dTPT3 and isoTAT). Overall, we were able to construct a multilevel DNA encryption system that, for the first time, introduces the innovations of UBP technology to combinatorially secure DNA-stored information.

Traditional encryption systems relying on computational schemes are vulnerable to brute-force attacks facilitated by supercomputers [50,51]. Moreover, they do not eliminate the possibility of leaking the information encoded by the natural four-letter (A/T/C/G) alphabet, as message-storing DNA can be easily decrypted by routine DNA sequencing [22,24,25]. The multilevel encryption system realized in this study provides a proof of principle for a framework combining the advances of both biotechnology and IT to comprehensively improve information security, providing an attractive multidimensional structure for data encryption (Fig. 5). Notably, the encryption system presented here is highly compatible with both *in vitro* and *in vivo* DNA storage formats, is amenable to commercial DNA synthesis and sequencing technology, and has no need for specialized instruments or reagents (dNaM/dTPT3/isoTAT only), making it an extraordinarily versatile scheme for encrypting DNA-encoded data. Specifically, DNA bearing dNaM-dTPT3 could be smoothly synthesized by commercial standard solid-phase oligonucleotide synthesis with an accuracy comparable with natural base pairs (more than 99.95%, as exemplified by the primers used in this work in Table S1), and could further be amplified by PCR with a natural base pair-like efficiency (only 4-fold lower than that of DNA containing just the natural base pairs) and with a fidelity of more than 99.98% [52]. Moreover, although the dNaM-dTPT3 pair could not be read directly via Sanger sequencing or other advanced technologies such as next-generation or third-generation sequencing, it could be converted into natural pairs of either G-C or T-A via transformed PCR depending on the presence or absence of the bridge base isoTAT to enable the UBP to be sequenced, which were shown to be successfully adapted for retrieving DNA sequences of a variety of UBP-containing DNAs using high-throughput sequencing [35]. Furthermore, with the use of portable DNA sequencing devices within reach, rapid data retrieval could be achieved, although error-correction strategies were required to handle the higher error rates [53]. Besides, UBP-containing DNAs could be successfully amplified from the DNA template stored for longer than 1 year (Fig. S6) and were previously shown as able to be propagated and retained with no detectable loss of accuracy in optimized *E. coli* strains during a long-term passaging experiment lasting 108 generations, demonstrating the long-term stability of UBP-containing DNAs stored *in vitro* or *in vivo* [35,54]. As a result, the present system provides a ‘proof of concept’ application of UBP to DNA encryption, which could serve as a useful reference for future improvements. It is also noteworthy that, by introducing UBPs for data storage, our approach might also achieve a high density of data storage, allowing us to encrypt data in a very small space. The IM-Codec algorithm compresses the running length of encoded DNA sequences with the aid of UBPs, consequently increasing the information density. It can reach an average tested theoretic information density of more than 2 bits/nt, with a maximum tested theoretic information density of 9.16 bits/nt, which should be superior to many previously reported systems [10,11,13,55]. Furthermore, in addition to the NaM-TPT3 pair, there are additional UBPs and several other modified nucleotides [56–58], such as hachimoji DNA, 5-methylcytosine (5mC), N_4_-methylcytosine (4mC) 5-formyluracil (5fU), 5-hydroxymethylcytosine (5hmC), 5-formylcytosine (5fC), 5-carboxylcytosine (5caC), 5-hydroxymethyluracil (5hmU) and N6-methyladenine (6mA), which can be produced, sequenced and potentially incorporated into our encryption system to further increase the storage density and potential for data encryption (Table S2) [30,31], while questions regarding orthogonality and stability, as well as the availability of reading and writing technologies, remain to be investigated in the future before their usage. Thus, the encryption system presented here has promise as a prototype and can be easily expanded to develop more complicated encryption systems by incorporating other UBPs or modified nucleotides. In addition, future work may incorporate DNA structure information (e.g. via DNA origami or a comparable structure) into our UBP encryption scheme to provide more robust data encryption [4]. Furthermore, we anticipate that other chemical forms of DNA (e.g. mirror DNA) may be paired with the UBP expanded genetic system to achieve large-scale data encryption within DNA [38].

Our multilevel DNA data encryption method may be useful for a range of vital applications (Fig. 5), such as storing private keys for bitcoin, bank card pins, personal health information and financial system information. The storage of confidential data in our system has various advantages: (i) the data are stored in a UBP-integrated DNA medium that is resistant to both computer hackers and routine sequencing; (ii) the data are physically separated by the KS and IS, providing additional protection; and (iii) the encrypted data stored by the UBP-based multilevel DNA encryption system should be tiny enough to be stored or carried securely or be replicated many times for long-term storage. We expect that future studies will focus on improving the system's utility in more practical scenarios. In conclusion, based on the introduction of UBPs, we developed a multilevel DNA data encryption system to enhance the security of data storage. Our system can offer a useful solution for practical DNA data storage that takes high privacy into account and supports the advancement of DNA encryption into an expanded genetic system-based era.

## METHODS

### UBP-containing DNA information synthesis

Oligonucleotides containing UB were synthesized by Sangon Biotech, and the phosphoramidite building blocks of UB were synthesized by ourselves and provided to Sangon Biotech. dNaMTP and dTPT3TP were synthesized according to previous methods [52]. The sequences of DNA information and oligonucleotides are detailed in Table S1. Next, 1 μM of each oligonucleotide was mixed with 25 μL of 2 × Hieff PCR Master Mix, 100 μM dNaMTP and dTPT3TP, and ddH_2_O, up to 50 μL. The DNA information sequences were synthesized by using the following thermal cycling conditions: 15 cycles of denaturation (94°C, 30 s), annealing (52°C, 30 s) and extension (72°C, 15 s), then a final extension (72°C, 10 min). The PCR products were examined and purified using a 1% agarose gel.

### DNA information library preparation and selectively amplifying

Primers for selectively amplifying are listed in Table S1. The indexed sequences for DNA information are incorporated through PCR, followed by mixing and storage after spin column purification. The target DNA sequence was amplified by PCR according to the following recipe: 25 μL of 2 × Hieff PCR Master Mix, 100 μM dNaMTP and dTPT3TP, 10 pg DNA information library, 1 μM Primers (with X, Y)-F, 1 μM Primers (with X, Y)-R, and ddH_2_O up to 50 μL. The thermocycling protocol was as follows: initial denaturation (94°C, 3 min), followed by 15 cycles of denaturation (94°C, 30 s), annealing (55°C, 30 s), and extension (72°C, 15 s), then a final extension (72°C, 10 min). Both forward and reverse index primers are employed for sequencing. The sequence of the DNA information is obtained following the splicing process.

### Transformed PCR and sequencing

Transformed PCR was performed as previously described [35] according to the following recipe: 25 μL 2 × Hieff PCR Master Mix, UBP-containing DNA information sequences, 1 μM each forward and reverse primer, and ddH_2_O up to 50 μL, Bridge base PCR add 100 μM disoTATTP and dNaMTP, inherent base's preference PCR add only 100 μM dNaMTP. Sequence both PCR products to identify the sequences of UBP-containing DNA information sequences.

### Second-generation and third-generation sequencing

DNAs bearing UBPs or not were amplified by PCR with or without the addition of dNaM-dTPT3 in the PCR reaction, and the resultant PCR products were sent for next-generation or third-generation sequencing. Next-generation sequencing was performed on the MGI2000 platform and quality control of the sequencing reads was performed according to the standard procedure of BGI Genomics. Third-generation sequencing was performed on the PacBio Sequel II platform and quality control of the sequencing reads was achieved following the standard protocol of Haorui Genomics. The resulting raw sequence data have been deposited in the NCBI BioProject (accession number: PRJNA1155232).

## Supplementary Material

nwae469_Supplemental_Files

## Data Availability

The data underlying this article will be shared upon reasonable request to the corresponding author.

## CODE AVAILABILITY

The code is available at https://github.com/DNAstorage-iSynBio/IM-Codec.git.
