# Supplementary material for: Towards next-generation DNA encryption via an expanded genetic system
Source: Natl Sci Rev. 2024 Dec 23;12(4):nwae469. doi: 10.1093/nsr/nwae469 (PMC11951100; doi:10.1093/nsr/nwae469)
Supplement: nwae469_Supplemental_Files [file nwae469_supplemental_files.zip › Supplementary Materials.pdf]

Supplementary Materials

Towards next-generation DNA encryption via an expanded genetic system

Xiaoluo Huang†*, Zhaohua Hou†, Wei Qiang†, Honglei Wang†, Xiangxiang Wang, Xiaoxu Chen, Xin Hu, Junbiao Dai*, Lingjun Li*, Guanghou Zhao*

Corresponding author: Guanghou Zhao*, zhaogh@nwpu.edu.cn; Xiaoluo Huang*, huangxl@siat.ac.cn; Lingjun Li*, lingjunlee@htu.cn; Junbiao Dai*, daijunbiao@caas.cn.

†These authors contributed equally to the paper.

Supplementary materials include:

Supplementary Figures

Supplementary tables

Supplementary Note

Supplementary Figures

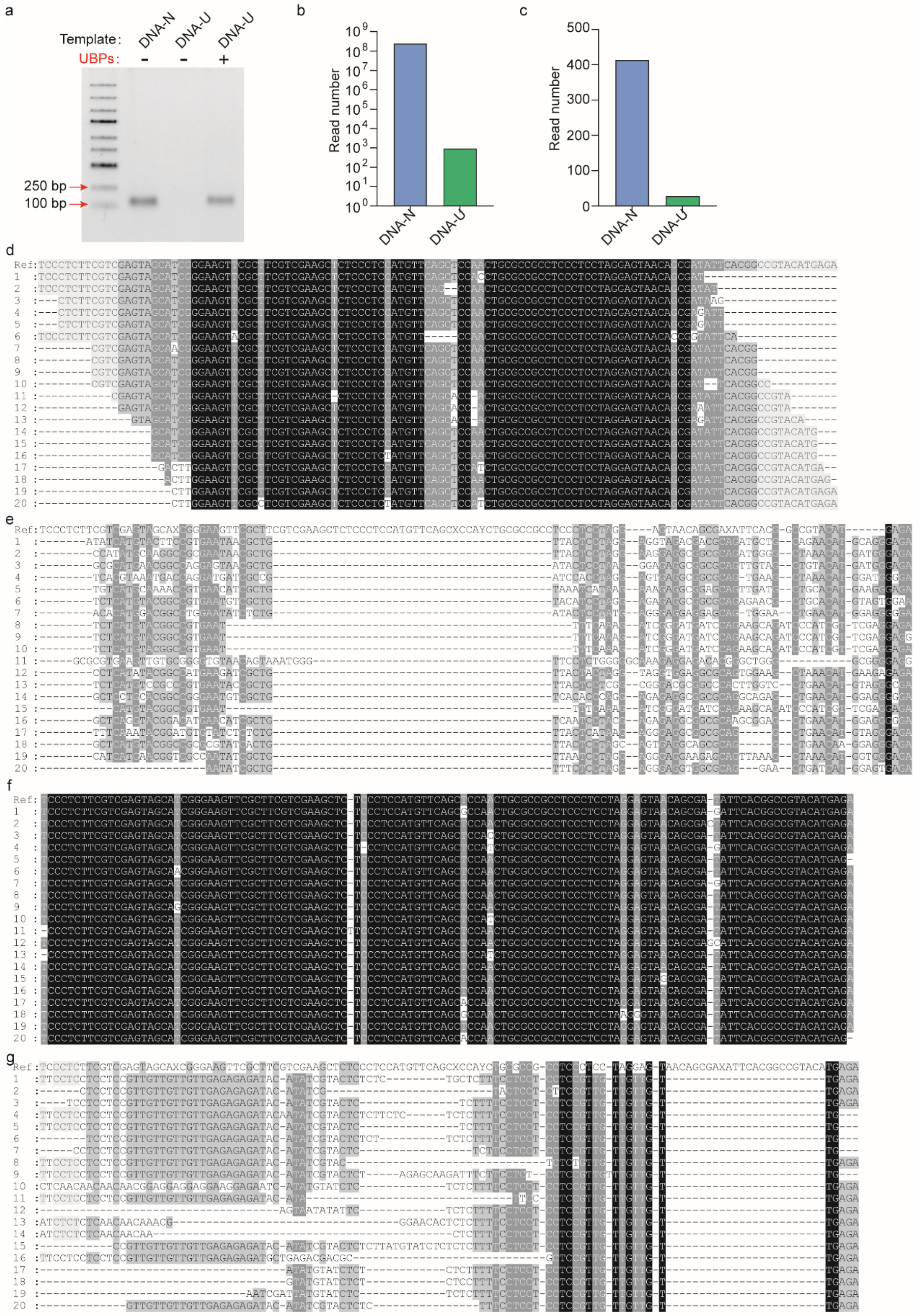

Figure S1. PCR, second-generation and third-generation sequencing analysis of DNA harboring UBPs or not. (a) Gel analysis of PCR products with (+) or without (-) the addition of unnatural deoxy-nucleoside triphosphates in the PCR reaction. DNA-N,

PCR products from the template DNA composed of only natural bases; DNA-U, PCR products from the template DNA harboring UBPs. (b, c) Summary of the read numbers of DNA-N and DNA-L obtained from the second-generation sequencing (b, performed on the MGI2000 platform) or third-generation sequencing (c, performed on the PacBio Sequel II platform). DNA-N, DNA composed of only natural bases; DNA-U, DNA harboring UBPs. (d, e) Sequence alignment of the reference sequences (marked as "Ref") of DNA-N (d) or DNA-U (e) with the DNA sequences obtained from second-generation sequencing (exemplified by 20 representative reads, marked as "1,2, 3, etc"). DNA-N, DNA composed of only natural bases; DNA-U, DNA harboring UBPs. (f, g) Sequence alignment of the reference sequences (marked as "Ref") of DNA-N (f) or DNA-U (g) with the DNA sequences obtained from third-generation sequencing (exemplified by 20 representative reads, marked as "1,2, 3, etc"). DNA-N, DNA composed of only natural bases; DNA-U, DNA harboring UBPs.

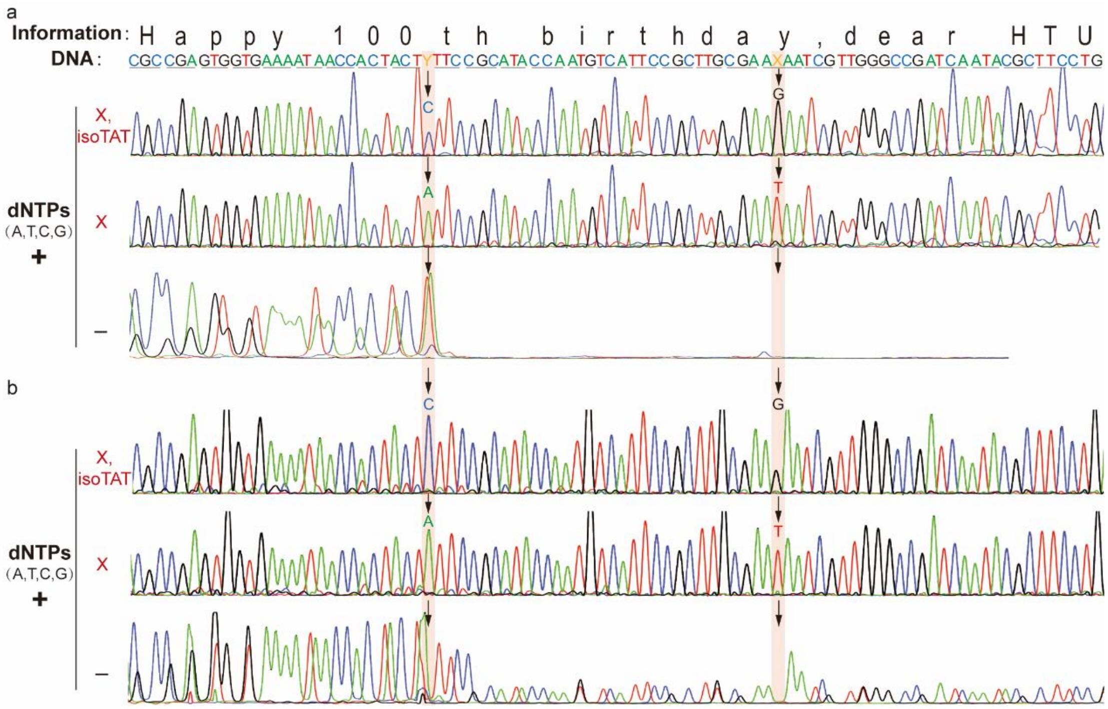

Figure S2. The sequencing peak diagram of the encrypted information "Happy 100th birthday,dear HTU" stored *in vitro* (a) and *in vivo* (b).

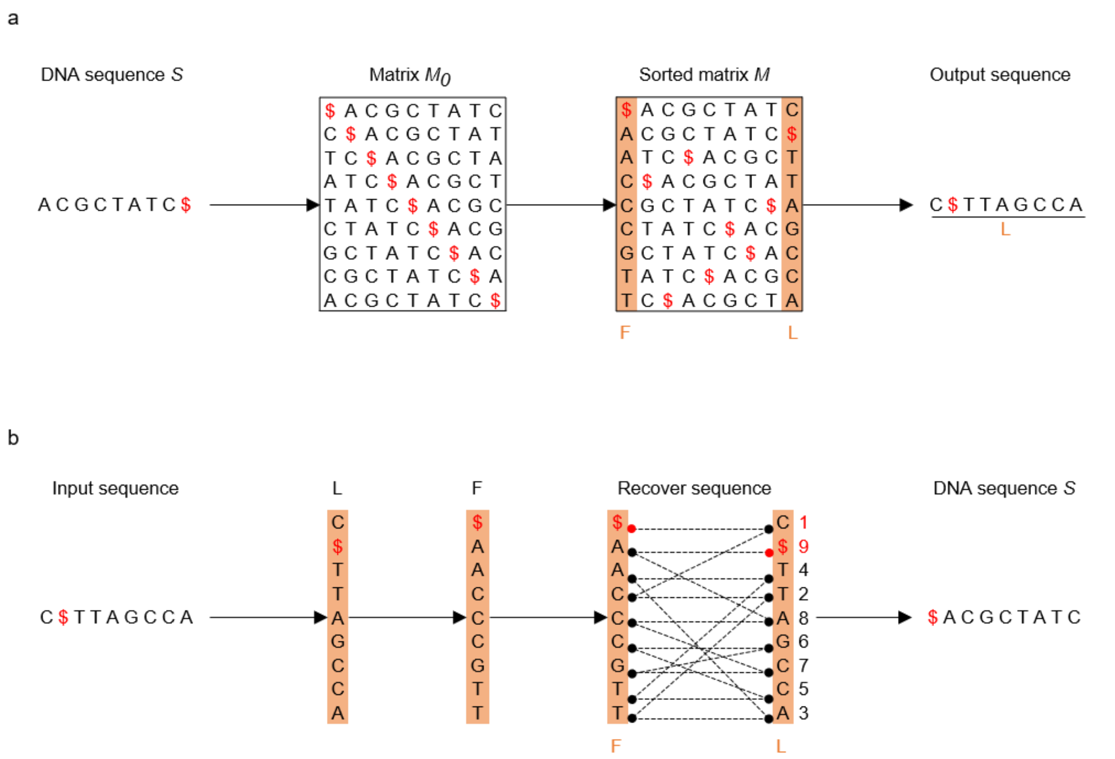

Figure S3. The detail process of BWT conversion. (a) The conversion process of BWT conversion. The Matrix “M0” and “M” are generated to produced sequence “L” as the output sequence. (b) The restoring process for BWT conversion. The sequence “L” is firstly converted into sequence “F”, which are then used to restore the original sequence.

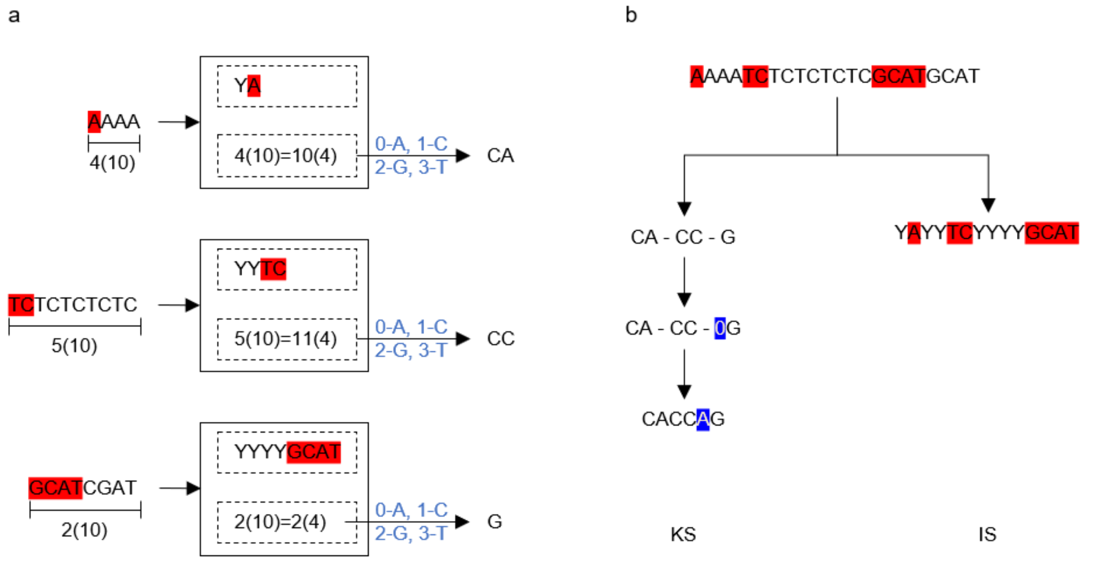

Figure S4. Encryption of different tandem repeats by IM-Codec algorithm. (a) Illustration of different tandem repeats conversion. Single nucleotide repeats, two nucleotide repeats and four nucleotide repeats were encrypted by IM-Codec conversion.

(b) Encryption of one sequence containing different types of tandem repeats into IS and KS sequence.

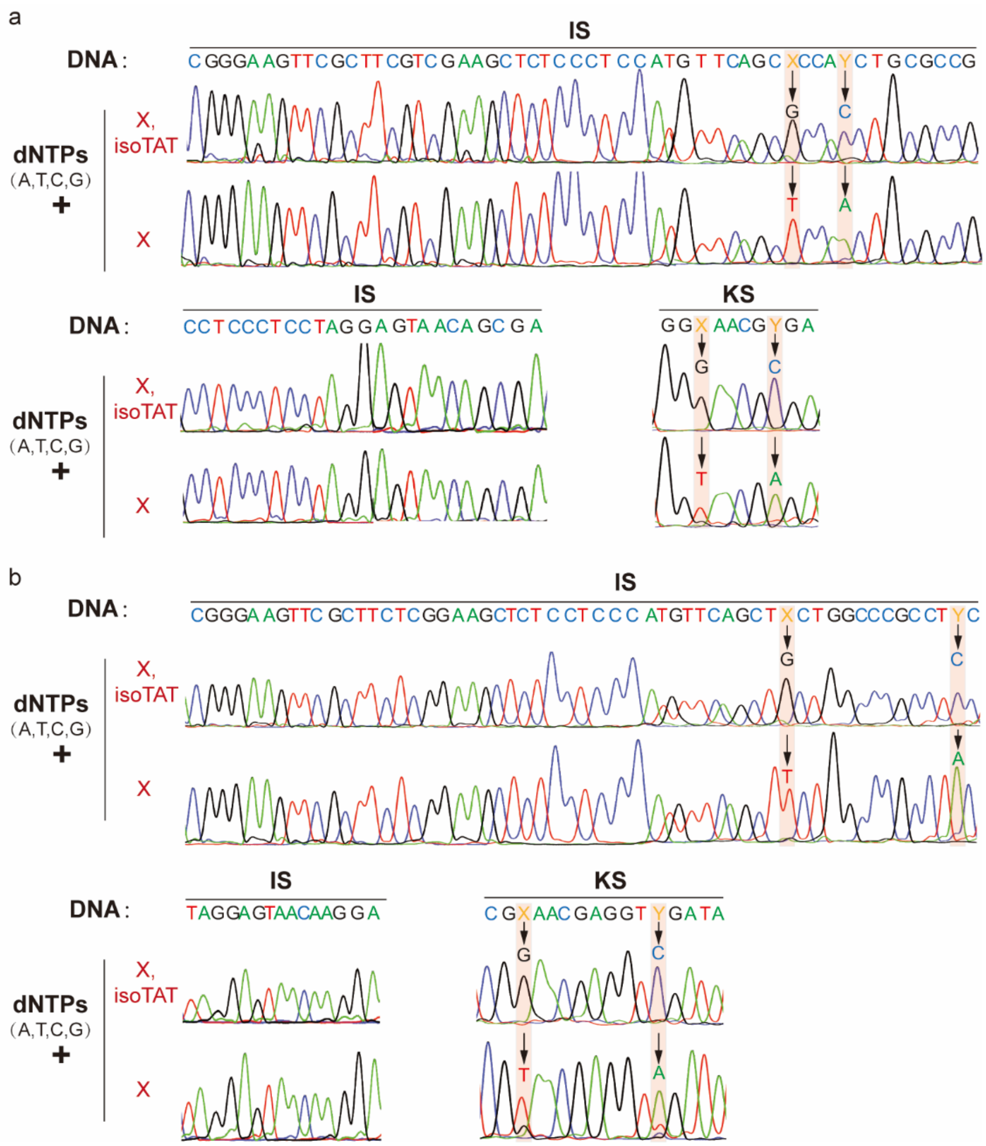

Figure S5. (a) IS and KS sequencing peak diagram of information "JUNE6 INVASION:NORMANDY". (b) IS and KS sequencing peak diagram of information "JUNE9 INVASION:NORMANDY".

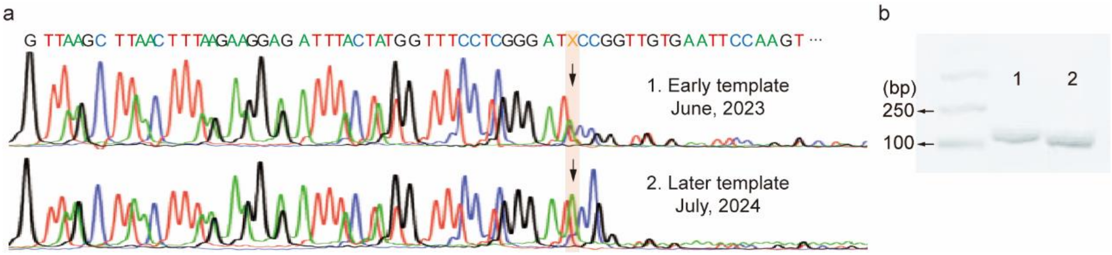

Figure S6. Stability analysis of DNA containing unnatural bases. Sanger sequencing of the PCR products from DNA templates undergoing different periods. Gel of PCR products from DNA templates undergoing different periods. 134-mer templates with NaM in DNA synthesized at June, 2023 (1) or July, 2024 (2) are used. PCR is performed with oligo-T primers for 36 cycles. Antisense stands are shown, and the purple arrowhead indicates the corresponding location of unnatural base.

Supplementary tables

Table S1 (separate file). Oligonucleotides used in this work.

Table S2 Encryption robustness analysis of IM-Codec algorithm

The encryption algorithm	The key information content	The number of brute force attacks required to decrypt the encrypted message	The number of brute-force attacks required to decrypt the encrypted message by IM-Codec with the same "key" information content
DES	2^{56}	2^{56}	$[2^{56}, \frac{4}{3} \times (2^{56} - 1)]$
AES	2^{256}	2^{256}	$[2^{256}, \frac{4}{3} \times (2^{256} - 1)]$
MD5	2^{128}	2^{128}	$[2^{128}, \frac{4}{3} \times (2^{128} - 1)]$
SHA-1	2^{160}	2^{160}	$[2^{160}, \frac{4}{3} \times (2^{160} - 1)]$
SHA-384	2^{384}	2^{384}	$[2^{384}, \frac{4}{3} \times (2^{384} - 1)]$
SHA-512	2^{512}	2^{512}	$[2^{512}, \frac{4}{3} \times (2^{512} - 1)]$

Table S3. The theoretic information density of data storage by IM-Codec algorithm[1]

File type	File name	File size (bytes)	Density (bits/nt)[2]		
			A/T/C/G+X/Y[3]	A/T/C/G+X/Y+4 additional UBPs/modified nucleotides[4]	A/T/C/G+X/Y+12 additional UBPs/modified nucleotides[5]
Text	PMC3618856.txt	10240.00	3.61	3.84	5.90
	PMC4070265.txt	10240.00	3.49	3.76	5.99
	PMC4147042.txt	10240.00	4.08	4.20	6.88
	PMC4147052.txt	10240.00	3.69	3.95	6.21
	PMC4147055.txt	10240.00	3.53	3.76	6.06
	PMC4365094.txt	10240.00	3.55	3.86	6.04
	PMC4406960.txt	10240.00	3.63	4.05	6.63
	PMC4406961.txt	10240.00	3.71	3.97	6.34
	PMC4706275.txt	10240.00	3.60	3.85	6.32
	PMC4706279.txt	10240.00	3.60	3.90	6.09
Image	1.jpg	7047.00	2.05	3.03	4.06
	10.jpg	7201.00	2.03	3.03	4.05
	2.jpg	10122.00	2.03	3.02	4.04
	3.jpg	9045.00	2.02	3.02	4.04
	4.jpg	10120.00	2.03	3.02	4.03
	5.jpg	10340.00	2.01	3.02	4.03
	6.jpg	9275.00	2.03	3.02	4.04
	7.jpg	9047.00	2.02	3.02	4.04
	8.jpg	10163.00	2.02	3.02	4.03

	9.jpg	7347.00	2.02	3.03	4.05
	flower.jpg	107387	2.00	3.00	4.01
	peacock.jpg	103807	2.00	3.00	4.01
	sky.jpg	108422	2.00	3.00	4.01
Audio	A_Little_Bit_Rhythm.mp3	10240.00	3.28	5.00	6.67
	A_Part_of_Us.mp3	10240.00	3.29	5.00	6.68
	Flying.mp3	10240.00	3.30	5.03	6.70
	in_the_memory.mp3	10240.00	2.16	3.27	4.37
	Only_the_Braves.mp3	10240.00	2.16	3.27	4.37
	Returning_Home.mp3	10240.00	3.28	5.01	6.68
	Spiritual.mp3	10240.00	3.30	5.02	6.69
	The_Wild_Side.mp3	10240.00	3.34	5.07	6.78
	What_Matters_Most.mp3	10240.00	2.04	3.06	4.09
	Wild_Flowers.mp3	10240.00	3.30	5.02	6.69
Video	2015-03-29-01.mp4	10240.00	3.04	4.39	6.00
	Adrift-HD.mp4	10240.00	5.61	7.78	10.64
	clouds.mp4	10240.00	3.12	4.12	6.02
	Cold.mp4	10240.00	4.34	5.87	8.58
	flow_in_the_sky.mp4	10240.00	3.36	4.54	6.54

Jellyfish.mp4	10240.00	2.82	4.05	5.54
MaunawiliFalls.mp4	10240.00	6.15	8.45	11.49
Nature.mp4	10240.00	**9.16**	11.63	17.29
OneBigCircle-HD.mp4	10240.00	3.98	5.58	7.67
PK-Dwyer-HD.mp4	10240.00	4.87	6.88	9.21
**Average density (bits/nt)**		3.22	4.34	6.13

1. A computer simulation is performed to calculate the theoretical information density of data storage using the IM-Codec algorithm using standard pipeline, similar to that shown in Figure 3. The theoretic information density represents the maximum information density that the codec system can achieve through *in silico* experiments, but it does not represent the practical information density demonstrated by wet-lab experiments, as current synthesis and sequencing technology of UBPs/modified nucleotides still require further large-scaled investigation.
2. The theoretic information density is calculated by dividing the size of data (bits) with the total amount of encoded nucleotides. Our algorithm encodes data directly into DNA sequences in a non-segmented manner. Consequently, the calculation of data density does not need to account for the addition of indices to multiple DNA fragments.
3. The theoretic information density is calculated while four natural A/T/C/G nucleotides are used for data encoding and X/Y is used for "IS" and "KS" conversion.
4. The theoretic information density is calculated while 4 additional UBPs/modified nucleotides are added into data encoding on top of the system developed at "3". All additional nucleotides are used for data encoding and X/Y is used for "IS" and "KS" conversion. 4 from 12 other reported unnatural base pairs [1,2] including P-Z, S-B and isoG and isoC and modified nucleotides [3,4] such as 5-methylcytosine (5mC), N_4-methylcytosine (4mC), 5-formyluracil (5fU), 5-formylcytosine (5fC), and 5-carboxylcytosine (5caC), N6-methyladenine (6mA) can be used for the analysis.
5. The theoretic information density is calculated while 12 additional UBPs/modified nucleotides are added into data encoding on top of the system developed at "3". All additional nucleotides are used for data encoding and X/Y is used for "IS" and "KS" conversion. 12 other reported unnatural base pairs [1,2] including P-Z, S-B and isoG and isoC and modified nucleotides [3,4]such as 5-methylcytosine (5mC), N_4-methylcytosine (4mC), 5-formyluracil (5fU), 5-formylcytosine (5fC), and 5-carboxylcytosine (5caC), N6-methyladenine (6mA) can be used for the analysis.

Table S4 The storage density variation under different IM-Codec parameters

Optional parameters		Density (bits/nt)[1]			
		Text	Image	Audio	Video
The number of nucleotides within every tandem repeat unit[2]	1	3.61	2.05	3.28	5.61
	2	3.47	2.03	3.35	5.62
	3	3.17	2.02	3.35	5.39
	4	2.96	2.02	3.35	5.22
	5	2.82	2.01	3.34	5.08
	6	2.72	2.01	3.34	5.00
	7	2.65	2.01	3.34	4.89
	8	2.58	2.01	3.34	4.76
	9	2.53	2.00	3.34	4.68
	10	2.46	2.00	3.34	4.60
The number of reduced "Y" bases[3]	5	3.61	2.04	3.28	5.59
	10	3.60	2.04	3.28	5.58
	15	3.60	2.04	3.27	5.57
	20	3.59	2.04	3.27	5.56
	25	3.59	2.03	3.26	5.55
	30	3.59	2.03	3.26	5.54
	35	3.58	2.03	3.25	5.53
	40	3.58	2.03	3.25	5.52
	45	3.57	2.02	3.24	5.50
	50	3.57	2.02	3.24	5.49
The count of repetitions for the tandem repeat unit[4]	1	2.41	1.35	1.47	3.08
	2	3.06	1.87	2.55	4.62
	3	3.41	2.02	3.12	5.29
	4	3.61	2.05	3.28	5.61
	5	3.72	2.05	3.34	5.75
	6	3.76	2.04	3.35	5.79

7	3.76	2.04	3.35	5.81
8	3.74	2.04	3.35	5.81
9	3.71	2.03	3.35	5.80
10	3.67	2.03	3.35	5.77

[1]Following Table S3, the theoretic information density is calculated while four natural A/T/C/G nucleotides are used for data encoding and X/Y is used for "IS" and "KS" conversion using IM-Codec algorithm with changed repeated units processing manners. Files of "PMC3618856.txt", "1.jpg", "A_Little_Bit_of_Rhythm.mp3", and "Adrift-HD.mp4" with sizes of 10, 240, 7, 047, 10, 240, and 10, 240 bytes were used for the analysis, respectively.

[2]"The number of nucleotides within every tandem repeat unit" refers to the number of nucleotides within tandem repeat unit that need to be converted, while the minimal count of repetitions for the tandem repeat unit are "4" and all "Y" bases are kept in the sequence.

[3]"NO. of reduced 'Y' base the number of 'Y' bases" that are removed from the IS, while the number of nucleotides within tandem repeat unit is "1" and the minimal count of repetitions for the tandem repeat unit are "4".

[4]"The count of repetitions for the tandem repeat unit" refers to the minimal count of repetitions for the tandem repeat unit in IS that need to be converted, while the number of nucleotides within tandem repeat unit is "1" and all "Y" bases are kept in the sequence.

Table S5 Mass spectral data for the DNA sequences bearing UBPs.

**Oligos Name**	**Mass Spectra (ESI)**	
	**Calc.(m/z)**	**Found (m/z)**
Message1-Encryption-1	12045.01	12046.4
Message1-Encryption-2	15797.46	15800.7
Message2-Encryption-1	12893.56	12894.3
Message2-Encryption-2	12960.61	12960.9

Message1-Add primer with X,Y-F	6380.35	6380.6
Message1-Add primer with X,Y-R	6453.40	6453.6
Message1-Primer with X,Y-F	17135.31	17137.9
Message1-Primer with X,Y-R	17673.65	17677.7
Message2-Add primer with X,Y-F	12656.43	12656.8
Message2-Add primer with X,Y-R	12699.45	12700.1
Message2-Primer with X,Y-F	6419.39	6419.7
Message2-Primer with X,Y-R	6455.43	6455.7
Message3-Add primer with X,Y-F	13267.83	13268.6
Message3-Add primer with X,Y-R	12949.65	12950.2
Message3-Primer with X,Y-F	6486.45	6486.8
Message3-Primer with X,Y-R	6424.42	6424.7
Message4-Add primer with X,Y-F	14215.48	14216.2
Message4-Add primer with X,Y-R	15201.07	15201.5
Message4-Primer with X,Y-F	6424.43	6424.9
Message4-Primer with X,Y-R	6477.43	6477.8
IS-1	16483.84	16485.2
IS-2	16996.32	17001.3
IS-3	15966.53	15969.2
KS-1	17319.44	17320.5
KS-2	17459.84	17466.1
Fake-IS-2	17448.64	17456.6
Fake-KS-2	17747.88	17756.3

Supplementary Note

1. Details of BWT conversion

The BWT algorithm is a data conversion algorithm that can put related characters in a sequence in neighboring locations, which has been widely used by next-generation sequencing data analysis software, including as BWA, Bowtie[1,2], Bowtie2[3,4], and others. Briefly, for the BWT conversion in this study, the DNA sequence is firstly

recorded as “S”, with each element in “S” ranging from a to Z. The identifier “ \$” is then inserted at the end of the sequence (in ASCII encoding, “ \$” is smaller than a and all other any elements from “S”). Then, a cyclic right shift (ROR) on “S” is performed, and for every nucleotide shift, a new sequence is generated. These sequences generated a matrix $M0$. All sequences are sorted from small to large, resulting in the matrix M, with the first sequence starting with \$. The sequence L formed by the last column of characters is the converted new sequence. Notably, in M, any vertical sequence element composition is the same as in "S". As a result, the constituent elements of L and the first column F are the same. Meanwhile, matrix M has been organized in such a way that F closely follows the sorting rule of small to large. While decoding, if the last column L of matrix M is known, the elements of L are sorted in ascending order, generating the sequence equal to the first column F of M. Furthermore, given that the sequence in M is obtained from S by ROR, the first and last characters of any sequence in M are adjacent in S, and the last character is the first character of another sequence. Therefore, in terms of aforementioned pattern, the sequence “S” can be restored by the combination of “F” and “L” at the help from the first character “ \$” of the first sequence.

2. Details for “repeats” conversion by IM-Codec algorithm

The IM-codec algorithm process the running length in the encoded sequence to encrypt sequence information and improve storage density.

Supposing that the repeating segment is denoted as “R”, and its repeating unit “U” can be denoted as:

$$R = U_1 U_2 \dots U_N \quad (1)$$

Supposing that the observed length of “U” is “n”, and then, the fragments utilized for replacement, R' , can be represented as:

$$R' = Z_1 Z_2 \dots Z_n U \quad (2)$$

where “Z” represented “marker base”. “IS” is then obtained by the replacement of R with R' in the original DNA sequence. However, given that “IS” does not keep the

repetition time “N” from “U”, this information is kept into another sequence, known as “KS”. Firstly, N is converted into a quaternary number, indicated as Q. Assuming that the DNA sequence has a total of “k” duplicate fragments, the quaternary string can be written as:

$$S_q = Q_1 Q_2 \dots Q_k \tag{3}$$

All quaternions have equal digits and are filled based on the set's maximal digit, m . Finally, KS can be obtained using the predefined mapping rules and “KS” has the following length:

$k \times m$.

The converted DNA sequence must meet the following conditions in the X/Y base encryption system:

$$\begin{cases} N > 4 \\ n = 1 \end{cases} \tag{4}$$

Using Y as the marker base, bind to Eq (2) to obtain:

$$R'_{XY} = YU \tag{5}$$

After BWT conversion, the end identifier $ is replaced by X base.

At this point, the “X” base and “Y” base are inserted into “IS” sequences.

3. Encryption robustness analysis of IM-Codec algorithm

In the IM-Codec algorithm, the length of the cryptographic key, KS, is determined by the number of converted SNH (Single Nucleotide Homopolymer) units derived from the original DNA sequence, as well as the maximum repetition count of the repeated SNH unit. During the decryption process, the number of converted SNH units can be calculated by counting the unnatural bases within IS. Nonetheless, in the absence of KS, calculating the maximum repetition count for SNH units from known IS becomes impractical. As a result, for a given IS, the length of the corresponding KS is integrally tied to the maximum repetition count of SNH unit repetition. When using a brute-force strategy to decode the IS, the uncertainty surrounding the critical parameter of the

repetition count necessitates an iterative approach, starting with a repetition count of one and gradually increasing, systematically exploring and deciphering the sequence. This makes the "KS" generated by IM-Codec more difficult to crack than that generated by conventional encryption schemes such as AES, DES, MD5, SHA-1, SHA-384, and SHA-512[5-11]. A detailed computational comparison is listed below.

We firstly calculated the number of brute-force attacks required for the IM-Codec algorithm following below formula. While "C" represents "the frequency of the X/Y marker in the IS", "N" represents "the length of the unit representing the repetition count in the KS (an unknown variable)", and "F" represents "the number of brute-force attacks", then,

$$\mathrm{F} = \sum_{i=1}^{N} (4^{C})^{i} \tag{6}$$

The above formula can be shortened to:

$$\mathrm{F} = \frac{4^{C}(1-4^{CN})}{1-4^{C}} \tag{7}$$

Conventional computer encryption systems, including AES, DES, MD5, SHA-1, SHA-384, and SHA-512, use keys of a predetermined length, and the complexity of decrypting these keys via exhaustive search grows with key length.

If we indicate the key length as l, the number of brute-force attacks would be:

$$\mathrm{F}_{\mathrm{conventional}} = 2^{l} \tag{8}$$

When the key information content of IM-Codec equals that of standard computer encryption methods, $l = 2CN$ and incorporating Eq. (8) yields:

$$\mathrm{F}_{\mathrm{conventional}} = 4^{CN} \tag{9}$$

By combining Eq. (7) and Eq. (9), we arrive at:

$$\frac{\mathrm{F}}{\mathrm{F}_{\mathrm{conventional}}} = \frac{4^{C+CN}-4^{C}}{4^{C+CN}-4^{CN}} \tag{10}$$

Since C, N are all positive integers, it follows that:

$$\frac{F}{F_{conventiaonal}} \geq 1 \quad (11)$$

Thus, when the keys have the same level of information content, IM-Codec requires more brute-force efforts to decipher the encrypted message than traditional computer encryption methods, including AES, DES, MD5, SHA-1, SHA-384, and SHA-512 do.

4. The synthesis and biochemistry assays for the amplification, measures of incorporation efficiency and fidelity.

The syntheses of the DNA templates were described previously[12]. Fully natural primers were purchased from Sangon Biotech (Shanghai, China). Reagents and solvents for the synthesis of unnatural templates and primers were obtained from WuXi AppTec (Wuxi, China) and/or Sigma-Aldrich company. The oligonucleotides were prepared using standard automated DNA synthesis with natural phosphoramidites and dNaM or dTPT3 phosphoramidites on controlled pore glass supports and an ABI Expedite 8905 synthesizer. After automated synthesis, the oligonucleotides were cleaved from the support, and deprotected by incubation in conc. aqueous ammonia overnight at room temperature, purified by DMT purification (glen-pak™ cartridge, Glen Research), and desalted over Sephadex G-25 (NAP-25 Columns, GE Healthcare). The concentration of single-stranded oligonucleotides was determined by UV absorption at 260 nm. All the synthesized DNA oligonucleotides bearing unnatural base pairs were identified by mass spectra (**Table S5**). No depurination errors were found in the synthesized DNA oligos containing UBPs under the described conditions.

PCR amplifications were performed in a total volume of 25 μL and with conditions specific for each assay as described previously (Wuyuan Zhu, et al. J. Am. Chem. Soc., 2022, 144, 44, 20165). After amplification, a 5 μL aliquot was analyzed on a 6% non-denaturing PAGE gel. The remaining solution was purified by spin-

column, followed by 2% agarose gel, recovered with Zymoclean Gel DNA Recovery Kit (Zymo Research), quantified by fluorescent dye binding (Quant-iT dsDNA HS Assay kit, Life Technologies), and sequenced on a 3730 DNA Analyzer (Applied Biosystems).

The DNA polymerase-mediate incorporation reaction efficiency, and the fidelity of TPT3-NaM unnatural base pair in PCR were measured in our previous work. Km/Vmax for this pair is 3.63x109[13]. The %fidelity of this pair is > 99.98% (the fidelity of dA-dT is 99.99% as the control)[14]. In all the sequencing data obtained in this work, no errors were found in both sites of natural bases and UBPs.

The unnatural nucleosides of NaM and TPT3 are commercially available (e.g. WuXi AppTec, ~ 1g/280 USD), or can be synthesized based on our previously reported[14]. Phosphoramidites NaM and TPT3, and triphosphates of NaM, TPT3 and isoTAT were synthesized as previously reported, based on the general procedures below." [13,14]. General DMTr-protection Procedure: To a solution of free nucleoside (~100 mg, 1eq.)and N,N-dimethylaminopyridine (0.2 eq.) in pyridine (1 mL), 4,4'-dimethoxytrityl chloride(1.3 eq.) and triethylamine (1.3 eq.) were added. The solution was stirred for 18 h atambient temperature. After quenching the reaction, the mixture wasevaporated to dryness in vacuum. The residue was extracted with EtOAc (50 mL x 3) and water (50 mL), and the organic layer was collected, dried over anhydrous Na2SO4, filtered,and evaporated to dryness under reduced pressure. The crude product was purified bysilica gel column chromatography (hexane:EtOAc = 3:1 to 2:1) to afford the DMTr-protected nucleoside as a white foam.

General Phosphoramidite Synthesis Procedure: To a solution of DMTr-protectednucleoside (100 mg, 1 eq.) and diisopropylethylamine (4 eq.) in dichloromethane (1mL) was added 2-cyanoethyl N,N-diisopropylchlorophosphoramidite (1.5 eq.) at 0 °C. Afterstirring for 2 h at ambient temperature, the reaction mixture was extracted with EtOAc(30 mL x 3) and saturated NaHCO3 aq. (20 mL). The organic layers were collected, driedover anhydrous Na2SO4, filtered, and evaporated to dryness under reduced pressure. Thecrude product was

purified by silica gel column chromatography (hexane:EtOAc = 3:1 to2:1) to afford compound as a pale yellow foam.

General Procedure for Triphosphate Synthesis: Proton sponge (1.5 equiv) and the free nucleoside (1 equiv) were dissolved in trimethyl phosphate (0.3 M) and cooled to -20 °C.POCl3 (1.5 equiv) was added dropwise, and the purple slurry was stirred at -20 °C for 2 h.Tributylamine (6.2 equiv) was added, followed by a solution of tributylammoniumpyrophosphate (5.0 equiv) in DMF (0.5 M). After 5 min, the reaction was quenched byaddition of 0.5 M aqueous Et3NH2CO3 and the resulting solution waslyophilized. Purification by reverse-phase (C18) HPLC (4-35% CH3CN in 0.1 MEt3NH2CO3, pH 7.5) followed by lyophilization afforded the triphosphate as a white solid.

5. UBP-Containing DNA information storage *in vivo*

UBP-Containing DNA information plasmid Constructions: The DNA information fragment incorporates the BsaI restriction recognition site through PCR according to the following recipe: 25 μL 2×Hieff PCR Master Mix, 100 μM dNaMTP and dTPT3TP, 0.1 ng DNA information fragment, 1 μM Encryption-F, 1 μM Encryption-R, and ddH_2O up to 50 μL. The thermocycling protocol under the following: initial denaturation (94 °C, 3 min); followed by 15 cycles of denaturation (94 °C, 30 s), annealing (55 °C, 30 s), and extension (72 °C, 15 s); and final extension (72 °C, 10 min). The vector fragment was amplified from modified pUC19 using primers pUC19-F and pUC19-R. The PCR product underwent spin column purification afterward. Then, the DNA information fragment was digested by BsaI, The vector fragment was digested by BsaI and DpnI. The digested product was purified using a 1% agarose gel. Two fragments are ligated using T4 ligase for 2 hours to obtain DNA information plasmid. The UBP-Containing DNA information plasmid sequence is shown in the

Supplementary Note 6.

UBP-Containing DNA information storage *in vivo:* Plasmid pACS2 carrying the ptNTT2 gene was introduced into the BL21(DE3) cells[14,15]. the transformants were plated onto the 2×YT-agar with 33 mg/L chloramphenicol, incubating at 37 ℃ overnight. Inoculate 3 ml of 2×YT medium containing 33 mg/L chloramphenicol and 50 mM KPi with the selected clone, incubating at 37 ℃ with shaking. The overnight culture was diluted 100-fold with the same selected medium, and growing to OD_{600} of 0.4 to 0.5. Cells were rapidly placed on ice for 10 minutes, then the cell was collected by spinning at 3500 rpm for 10 minutes at 4 ℃ and washed three times with 30 ml of ice-cold 15% glycerol. Resuspend the cells in 1ml of ice-cold 15% glycerol and dispense in 50 μL aliquots. The aliquot of cells was mixed with 2 μL of UBP-Containing DNA information plasmid and transferred to a prechilled 0.2 cm gap electroporation cuvette without introducing bubbles. Electroporate using the following conditions for Bio-Rad MicroPulser Electroporator: Ec2, voltage 2.5 kV. Immediately add 400 μL of 2×YT medium containing 50 mM KPi, 125 μM dNaMTP, and 125μM dTPT3TP, and recover for 1 hour at 37 ℃ with shaking at 220 rpm. After recovery, the cell pellet was collected and resuspended in 100 μL of 2×YT medium containing 5 mg/L chloramphenicol, 50 mg/L ampicillin, 50 mM KPi, 125 μM dNaMTP, and 125 μM dTPT3TP. The cells carrying UBP-Containing DNA information plasmid were grown at 37 ℃ for 17 hours with shaking at 220 rpm. For reading, take 1 μL of the bacterial solution as a template and utilize M13F/R to perform The Transformed PCR and sequence.

6. BP-Containing DNA information plasmid sequence

AGTCTGTCTGGCTTAATAAYTGTCTCCTCGAACGATGGGXATCTGCTTCTGGATCATCC
CGATCTTTGAAAattcTgagacCCCTGCAGGATAACTTCGTATAGCATACATTATACGAAGTT
ATcgttAGAGACGGAGTCACTGCCAACCGAGACGGTCATAGCTGTTTCCTGTGTGCCGCT
TCCTCGCTCACTGACTCGCTGCGCTCGGTCGTTCGGCTGCGGCGAGCGGTATCAGCTC
ACTCAAAGGCGGTAATACGGTTACCCACAGAATCAGGGGATAACGCAGGAAAGAACA
TGTGAGCAAAAGGCCAGCAAAAGGCCAGGAACCGTAAAAAGGCCGCGTTGCTGGCG
TTTTTCCATAGGCTCCGCCCCCCTGACGAGCATCACAAAAATCGACGCTCAAGTCAGA
GGTGGCGAAACCCGACAGGACTATAAAGATACCAGGCGTTTCCCCCTGGAAGCTCCCT
CGTGCGCTCTCCTGTTCCGACCCTGCCGCTTACCGGATACCTGTCCGCCTTTCTCCCTT
CGGGAAGCGTGGCGCTTTCTCATAGCTCACGCTGTAGGTATCTCAGTTCGGTGTAGGTC
GTTCGCTCCAAGCTGGGCTGTGTGCACGAACCCCCCGTTCAGCCCGACCGCTGCGCCT
TATCCGGTAACTATCGTCTTGAGTCCAACCCGGTAAGACACGACTTATCGCCACTGGCA
GCAGCCACTGGTAACAGGATTAGCAGAGCGAGGTATGTAGGCGGTGCTACAGAGTTCT
TGAAGTGGTGGCCTAACTACGGCTACACTAGAAGGACAGTATTTGGTATCTGCGCTCTG
CTGAAGCCAGTTACCTTCGGAAAAAGAGTTGGTAGCTCTTGATCCGGCAAACAAACCA
CCGCTGGTAGCGGTGGTTTTTTTGTTTGCAAGCAGCAGATTACGCGCAGAAAAAAAGG
ATCTCAAGAAGATCCTTTGATCTTTTCTACGGGGTCTGACGCTCAGTGGAACGAAAAC
TCACGTTAAGGGATTTTGGTCATGAGATTATCAAAAAGGATCTTCACCTAGATCCTTTTA
AATTAAAAATGAAGTTTTAAATCAATCTAAAGTATATATGAGTAAACTTGGTCTGACAGT
TACCAATGCTTAATCAGTGAGGCACCTATCTCAGCGATCTGTCTATTTCGTTCATCCATA
GTTGCCTGACTCCCCGTCGTGTAGATAACTACGATACGGGAGGGCTTACCATCTGGCCC
CAGTGCTGCAATAATACCGCGGGACCCACGCTCACCGGCTCCAGATTTATCAGCAATAA
ACCAGCCAGCCGGAAGGGCCGAGCGCAGAAGTGGTCCTGCAACTTTATCCGCCTCCAT
CCAGTCTATTAATTGTTGCCGGGAAGCTAGAGTAAGTAGTTCGCCAGTTAATAGTTTGC
GCAACGTTGTTGCCATCGCTACAGGCATCGTGGTATCACGCTCGTCGTTTGGTATGGCT
TCATTCAGCTCCGGTTCCCAACGATCAAGGCGAGTTACATGATCCCCCATGTTGCGCAA
AAAAGCGGTTAGCTCCTTCGGTCCTCCGATCGTTGTCAGAAGTAAGTTGGCCGCCGTG
TTATCACTCATGGTTATGGCAGCACTACATAATTCTCTTACTGTCATGCCATCCGTAAGAT
GCTTTTCTGTGACTGGTGAGTACTCAACCAAGTCATTCTGAGAATAGTGTATGCGGCGA
CCGAGTTGCTCTTGCCCGGCGTCAATACGGGATAATACCGCGCCACATAGCAGAACTTT
AAAAGTGCTCATCATTGGAAAACGTTCTTCGGGGCGAAAACTCTCAAGGATCTTACCG
CTGTTGAGATCCAGTTCGATGTAACCCACTCGTGCACCCAACTGATCTTCAGCATCTTT
TACTTTCACCAGCGTTTCTGGGTGAGCAAAAACAGGAAGGCAAAATGCCGCAAAAAA
GGGAATAAGGGCGACACGGAAATGTTGAATACTCATACTCTTCCTTTTTCAATATTATTG
AAGCATTTATCAGGGTTATTGTCTCATGAGCGGATACATATTTGAATGTATTTAGAAAAA
TAAACAAATAGGGGTTCCGCGCACATTTCCCCGAAAAGTGCCACCTGACGTCTAAGAA
ACCATTATTATCATGACATTAACCTATAAAAATAGGCGTATCACGAGGCCCTTTCGTTGT
AAAACGACGGCCAGTCGAACCACGCAATGCGTCTCGATCCGCAGTGTCTTGCGTCTCT
ggtgATAACTTCGTATAGCATACATTATACGAAGTTATactagtggGgtctcgtcta

Reference:

1. Hoshika S, Leal NA, Kim MJ et al. Hachimoji DNA and RNA: A genetic system with eight building blocks. Science. 2019; 363(6429): 884-887. doi: 10.1126/science.aat0971
2. Hirao I, Kimoto M, Yamashige R. Natural versus artificial creation of base pairs in DNA: origin of nucleobases from the perspectives of unnatural base pair studies. Acc Chem Res. 2012; 45(12): 2055-2065. doi: 10.1021/ar200257x
3. Chen X, Xu H, Shu X et al. Mapping epigenetic modifications by sequencing technologies. Cell Death & Differentiation. 2023. doi: 10.1038/s41418-023-01213-1
4. Dai Y, Yuan B-F, Feng Y-Q. Quantification and mapping of DNA modifications. RSC Chemical Biology. 2021; 2(4): 1096-1114.
5. Heron S. Advanced Encryption Standard (AES). Network Security. 2009; 2009(12): 8-12. doi: https://doi.org/10.1016/S1353-4858(10)70006-4
6. Joan Daemen VR. The design of Rijndael: AES-the advanced encryption standard[J]. Information Security and Cryptography2002.
7. Data Encryption Standard (DES). National Bureau of Standards1977.
8. Coppersmith D, Matyas, S. M., & Meyer, C. J. . DES (Data Encryption Standard). IBM Journal of Research and Development. 1978; 22(4): 289-294.
9. Rivest R. RFC1321: The MD5 Message-Digest Algorithm: RFC Editor, 1992.
10. Secure Hash Standard. National Institute of Standards and Technology. 1995; 180-1.
11. Secure Hash Standard (SHS). National Institute of Standards and Technology. 2012.
12. Zhu W, Wang H, Li X et al. Amplification, Enrichment, and Sequencing of Mutagenic Methylated DNA Adduct through Specifically Pairing with Unnatural Nucleobases. J Am Chem Soc. 2022; 144(44): 20165-20170. doi: 10.1021/jacs.2c06110
13. Wang H, Zhu W, Wang C et al. Locating, tracing and sequencing multiple expanded genetic letters in complex DNA context via a bridge-base approach. Nucleic Acids Research. 2023; 51(9): e52-e52. doi: 10.1093/nar/gkad218
14. Li L, Degardin M, Lavergne T et al. Natural-like replication of an unnatural base pair for the expansion of the genetic alphabet and biotechnology applications. J Am

Chem Soc. 2014; 136(3): 826-829. doi: 10.1021/ja408814g
15. Zhang Y, Lamb BM, Feldman AW et al. A semisynthetic organism engineered for the stable expansion of the genetic alphabet. Proc Natl Acad Sci U S A. 2017; 114(6): 1317-1322. doi: 10.1073/pnas.1616443114
